# Epigenetic Therapy for Breast Cancer

**DOI:** 10.3390/ijms12074465

**Published:** 2011-07-11

**Authors:** Feng-Feng Cai, Corina Kohler, Bei Zhang, Ming-Hong Wang, Wei-Jie Chen, Xiao-Yan Zhong

**Affiliations:** 1 Laboratory for Gynecological Oncology, Department of Biomedicine, Women’s Hospital, University of Basel, Hebelstrasse 20, Room 420, Basel, CH 4031, Switzerland; E-Mails: fcai@uhbs.ch (F.-F.C.); kohlerc@uhbs.ch (C.K.); zhangb@uhbs.ch (B.Z.); wchen@uhbs.ch (W.-J.C.); 2 Department of General Practice Medicine, Zhongda Hospital of Southeast University, Nanjing 210009, Jiangsu, China; E-Mail: wangminghong@medmail.com.cn

**Keywords:** breast cancer, epigenetic therapy, DNA methylation inhibitors, Histone deacetylation inhibitors

## Abstract

Both genetic and epigenetic alterations can control the progression of cancer. Genetic alterations are impossible to reverse, while epigenetic alterations are reversible. This advantage suggests that epigenetic modifications should be preferred in therapy applications. DNA methyltransferases and histone deacetylases have become the primary targets for studies in epigenetic therapy. Some DNA methylation inhibitors and histone deacetylation inhibitors are approved by the US Food and Drug Administration as anti-cancer drugs. Therefore, the uses of epigenetic targets are believed to have great potential as a lasting favorable approach in treating breast cancer.

## 1. Introduction

Breast cancer is one of the most common cancers among women [1]. Although early detection and improved treatment have increased breast cancer survival rates during the last decade, the 10 year survival rate is still about 80% [2]. This shows that there is still need for developing novel therapeutic strategies.

Genetic and epigenetic alterations have both been shown to play an important role in a variety of cellular processes which include chromatin remodeling, imprinting, X chromosome inactivation, and carcinogenesis [3]. The most common types of epigenetic alterations include DNA methylation and histone modifications. Unlike genetic mutations, epigenetic changes are reversible making them a more promising and safer alternative in breast cancer therapy.

The treatments of breast cancer demand multidisciplinary therapies. The state-of-the-art treatment options usually include a combination of surgery therapy, radiation therapy, cytotoxic chemotherapy, and molecularly targeted endocrine therapy depending on the type of breast cancer diagnosed [4]. During recent years, a lot of effort has been put into improving targeted therapy, in particular the following two therapies: trastuzumab (Herceptin), directed against the human epidermal growth factor receptor 2 (HER2); and bevacizumab, directed against vascular endothelial growth factor (VEGF). Both targeted therapies have been approved as milestones [5].

Recently, new treatment strategies focusing on epigenetic alterations have been suggested over gene mutation because of reversibility. The establishment and maintenance of epigenetic modifications rely on the operations of special enzymes, DNA methyltransferases and histone deacetylases, which have become the primary targets for epigenetic therapy [6,7]. Epigenetic therapies using the inhibitors of these enzymes have anti-tumorigenic effects on malignant conditions [8]. Therefore, this review will mainly focus on DNA methylation inhibitors and histone deacetylation inhibitors evaluating their potential for future application in epigenetic therapy.

## 2. DNA Methylation Inhibitors (DNMT Inhibitors)

DNA methylation in regulatory regions of genes has been shown to influence gene expression. 5-hydroxymethylcytosine (5hmC), a novel DNA modification in mammalian genomic DNA, can lead to demethylation of DNA and may contribute to the dynamics of DNA methylation [9,10]. Hypermethylation at GpG islands has been linked to transcriptional inactivation of genes. In cancer, these instances of hypermethylation often can be found in genes’ promoter regions, which are involved in cell cycle regulation, apoptosis, DNA repair, and informally known as tumor suppressor genes. DNA hypermethylations at CpG islands have been found in a variety of malignancies including acute myelogenous leukemia (AML), myelodysplastic syndrome (MDS) and other malignancies [11,12].

DNA methylation patterns are established and maintained by a family of enzymes called DNA methyltransferases [13]. During the methylation process, these enzymes catalyze the transfer of a methyl group to the 5 position of cytosin. In mammals, there are three active human DNA methyltransferases, DNMT1, DNMT3A, and DNMT3B. DNMT1 has importance in post-replicative maintenance of DNA methylation patterns in mammalian cells. DNMT3A and DNMT3B, two closely related enzymes, are considered to play a critical role in the de novo establishment of methylation patterns [14]. DNA demethylation can be achieved either by the failure of maintenance methylation after DNA replication, or by replication-independent processes involving base excision repair (BER) and nucleotide excision repair (NER) [15,16]. Tahiliani *et al.* suggested that the enzyme TET1, an iron-dependent a-ketoglutarate dioxygenase, may be responsible for the conversion of 5-methylcytosine (5mC) to 5-hydroxymethylcytosine (5hmC). It provided potential possibilities for demethylation [9].

Recent drug developments mainly focus on DNMT inhibitors (DNMTi) including nucleoside analogues and non-nucleoside analogues. By inhibiting DNMTs, genes that might have been silenced by DNA methylation in the course of the carcinogenic process could be reactivated, and the non-carcinogenic status of the cell could be reconstituted. The advantages of DNMT inhibitors are that they are not cancer type specific and could be used to treat various cancers [17].

### 2.1. Nucleoside Analogues

Nucleosides analogues are inhibitors of DNA synthesis and imputed in direct or indirect regulation of DNA methylation [18]. The mechanism of action in nucleoside analogues is based on their transformation to nucleotides and their subsequent incorporation into DNA. The formation of covalent complexes with DNMTs results in enzyme depletion and finally, a reversal of the methylation pattern [19]. There are four well-characterized nucleoside analogue methylation inhibitors, 5-azacytidine, 5-aza-2′-deoxycytidine (5-Aza-CdR), 5′-fluoro-2′-deoxycytidine and Zebularine.

#### 2.1.1. 5-Azacytidine

5-azacytidine (5-Aza-CR; Vidaza; azacitidine), a global DNMTi, was approved by FDA for the treatment of myelodysplastic syndrome (MDS). The clinical trials that use this product against different solid tumors have been carried out [20]. Azacitidine has two mechanisms of antineoplastic action—cytotoxicity and DNA demethylation [21]. It can be incorporated into both DNA and RNA. 5-Aza-CR treatment of mammalian cells also leads to defective tRNAs and rRNAs, and inhibits protein synthesis [22]. It is considered to cause chromosomal rearrangements and contribute to cytotoxicity [23].

#### 2.1.2. 5′-Aza-2′-Deoxycytidine

5′-aza-2′-deoxycytidine (5-azaCdR; DAC; decitabine), a cytosine analogue, is also incorporated into DNA during replication. 5′-aza-2′-deoxycytidine inhibits both DNMT1 and DNMT3B. It also leads to enhanced acetylation of histones H3 and H4 at the promoter regions. The use of the activating histone mark dimethylated lysine 4 of H3 was found to be enhanced by DAC by modulating gene expression [24]. 5′-aza-2′-deoxycytidine activates both silenced tumor suppressor genes and pro-metastatic genes by demethylation [20]. PDZ-LIM domain-containing protein 2 (PDLIM2) contains a tumor suppression function and has been shown to be repressed in breast cancer cells. The treatment of breast cancer cells with 5-aza-2′-deoxycytidine reversed the methylation of the PDLIM2 promoter, restored PDLIM2 expression, and suppressed tumorigenicity of human breast cancer cells [25].

5-aza-CdR induces tumor necrosis factor-related apoptosis-inducing ligand (TRAIL) in human breast cancer MDA-231 cells [26]. 5-aza-CdR is pivotal in enhancing chemosensitivity of breast cancer cells to anticancer agents [27].

#### 2.1.3. 5′-Fluoro-2′-Deoxycytidine

The nucleoside analogue 5′-fluoro-2′-deoxycytidine (5-F-dC; 5-F-CdR) is being evaluated clinically as a DNA methyltransferase inhibitor. It has an inhibitive effect on the action of the methyl transfer reaction [28,29]. However it has a lesser underlying effect as a drug since it leads to potentially toxic products [30].

#### 2.1.4. Zebularine

Zebularine is characterized as an inhibitor of cytidine deaminase with antitumor characteristics inhibiting DNA methylation and reactivating silenced genes similarly to 5-aza-CdR. The mechanism of action of zebularine as a DNMTi also requires incorporation into DNA after phosphorylation of zebularine to the diphosphate level and conversion to a deoxynucleotide [31]. It acts through post-transcriptional inhibition of DNMTs, inhibition of methyl CpG binding proteins, and alteration of global histone acetylation status. In contrast to other DNMTi, Zebularine is relatively less toxic to breast cancer cell lines [32]. The ability to manage zebularine with other epigenetic therapeutics with the least additive effect has also been established. Zebularine has antimitogenic and angiostatic activities [33].

### 2.2. Non-Nucleoside Analogues

A few non-nucleoside analogues are known to inhibit DNA methylation and rarely made it to clinical trials but active research in this field will possibly lead to the introduction of more compounds of this class in the near future. Non-nucleoside analogues inhibit DNA methylation by binding directly to the catalytic region of the DNMT without incorporating into DNA [34].

RG108, was first characterized by Brueckner *et al.* in 2005. They showed that it effectively prevented DNA methyltransferases *in vitro* in human cell lines. It causes demethylation and reactivation of tumor suppressor genes while not affecting the methylation pattern of centromeric satellite sequences [35]. So far, RG108 has not yet entered clinical trials.

Epigallocatechin-3-gallate (EGCG) is the main polyphenol compound of green tea. Treating cancer cells with micromolar concentrations of EGCG showed reduced DNA methylation and elevated transcription of tumor suppressor genes [36]. EGCG is currently being tested in Phase I trials and will be evaluated in phase II and III trials in the near future [37,38].

Psammaplins are derived from the sponge Psseudoceratina purpurea. They are inhibitors of both DNMTs and HDACs [39]. NVP-LAQ824, a Psammapalin derivative, has shown antitumor activity in preclinical studies [40]. It is currently undergoing Phase I clinical trials for hematologic malignancies.

MG98 is an antisense oligonucleotide which prevents translation of DNMT1 mRNA by hybridizing to the 3′ untranslated region of the DNMT1 mRNA. In addition to MG98’s great advantage due to its low toxicity, MG98 also demonstrates no antitumor activity in various solid cancers and no dose-related effects in Phase I studies; however, a Phase II study is currently underway [41,42].

The cardiovascular drug, hydralazine, promotes demethylation and tumor suppressor gene transcriptional reactivation. Hydrazine is also most likely to increase the efficacy of current biological or chemotherapeutic treatments [43]. In Phase I clinical trials, the drug has been shown to be well-tolerated and devoid of the common side effects of cytotoxic chemotherapy agents. Zambrano *et al.* additionally showed up to 52% demethylation of promoter regions in selected tumor suppressor genes upon treatment with different dose levels of the compound. A phase II clinical study using hydralazine in combination with standard cytotoxic chemotherapy is being planned as proof of concept that the reactivation of tumor suppressor genes silenced by DNA methylation increases chemotherapy efficacy in solid tumors [44].

## 3. Histone Deacetylation Inhibitors (HDAC Inhibitors)

Histone deacetylation inhibitors (HDAC inhibitors) inhibit histone deacetylase enzymes leading to the accumulation of acetylation in histones and then changing cellular processes that have become defective in cancerous cells. They have been shown to accumulate hyper-acatetylated histones and inhibit tumors [45]. HDAC inhibitors can be divided into four groups: shortchain fatty acids, hydroxamic acids, cyclic tetrapeptides and benzamides [46]. In humans, there are 11 zinc-dependent HDAC inhibitors. Different classes of HDAC inhibitors are now in clinical development for the treatment of both hematologic and solid tumors. Isoform selective HDAC inhibitors in combination with anti-cancer agents may serve as a future strategy for breast cancer therapy [47].

### 3.1. Shortchain Fatty Acids

Butyrate was the first HDAC inhibitor that was shown to inhibit cell growth and induce apoptosis [48]. Butyrate causes hyperacetylation of H3 and H4. Sodium butyrate can enhance radiosensitivity in MCF-7 breast cancer cell lines and can trigger apoptosis by the induction of caspase-10 expression [49]. Valproic acid (VPA), a well-tolerated antiepileptic drug with anti-tumor effects, is the only clinically available histone deacetylase inhibitor on both estrogen-sensitive and estrogen-insensitive breast cancer cells. VPA is a powerful antiproliferative agent in estrogen-sensitive breast cancer cells, making this drug of clinical interest as a new approach in treating breast cancer. Valproic acid inhibits HDAC activity *in vitro* and *in vivo*, and relieves HDAC-dependent transcriptional repression and causes hyperacetylation of histones. It is most likely that the acid achieves this through binding to the catalytic center of HDACs. Valproic acid induces differentiation of carcinoma cells. Tumor growth and metastasis formation have been shown to be significantly reduced in animal experiments [50]. Valproic acid induces proteasomal degradation of HDAC2 by selectively inhibiting the catalytic activity of class I HDACs. It induces ERα mRNA and protein without modifying ERβ in breast cancer cells. It reprograms the cells to a more differentiated and physiologic phenotype in both ERα-positive and ERα-negative malignant mammary epithelial cells that can improve the sensitivity to endocrine therapy and chemotherapy in breast cancer patients [51].

### 3.2. Hydroxamic Acids

Hydroxamates are active at micromolar to subnanomolar concentrations. Trichostatin A (TSA) is the first hydroxamic acid HDAC inhibitor identified, and most efficiently alters breast cancer cell viability. TSA, which is derived from *Streptomyces*, possesses anti-HDAC activity. The effect of TSA on cell proliferation and differentiation can be attributed to the inhibition of HDAC. TSA inhibits growth of ERα-positive breast cancer cells *in vitro* and also inhibits breast tumor growth *in vivo.* TSA enhances acetylation as well as the stability of the ERα protein and p300 protein; proteins that may contribute to the treatment of human breast cancer [52]. TSA synergizes with the demethylating agent 5-Aza-CdR in the re-expression of genes silenced in the process of carcinogenesis. Additionally, TSA combined with EGCG has been shown to have the synergistic effect of reactivating ERα expression in ERα-negative breast cancer cells [53].

Suberoylanilide hydroxamic acid (SAHA) is a small molecule inhibitor of both class I and II HDAC enzymes that inhibits the biological target *in vivo*, and has antitumor activity in solid and hematological tumors. SAHA has been approved by the FDA for the treatment of cutaneous manifestations of cutaneous T-cell lymphoma in patients [54]. It can be safely administered in a chronic oral treatment. SAHA has been investigated in phase I clinical trials in hematological and non-hematological malignancies. Several phase II studies of SAHA are ongoing in breast and other solid malignancies [55]. SAHA increases the anti-tumor effects of taxol in breast cancer *in vitro* and *in vivo* by analyzing the cell cycle and apoptosis. It indicates that the synergistic effects result from enhanced G2/M arrest and apoptosis. It also interferes with apoptotic pathway activities. The co-treatment, SAHA and trastuzumab or docetaxel, induces synergistic cytotoxic effects against breast cancer cells [56].

### 3.3. Cyclic Tetrapeptides

Trapoxin (TPX) has been shown to cause accumulation of highly acetylated core histones in a series of mammalian cell lines [57]. Low concentrations of TPX inhibit deacetylation of acetylated histone molecules irreversibly. TPX has not shown any effects in animal models because of its metabolic instability *in vivo* and has therefore also not come to clinical use [58]. Trapoxin binds covalently to the histone deacetylase through the epoxide moiety. Trapoxin induces the biological effects on the cell cycle and differentiation. TPX, an inhibitor of the eukaryotic cell cycle and inducer of morphological reversion of transformed cells, inhibits histone deacetylase at nanomolar concentrations.

Depsipeptide (FK228, FR901228), a bicyclic peptide isolated from Chromobacterium violaceum, containing a non-cystine disulfide bridge, increases the expression of lipoplex-delivered genes in cultured tumor cells. Depsipeptide increases the expression of the human p53 gene in metastatic breast cancer cells, but not in adjacent normal cells [59]. It can be safely administered when given as a 4-hour infusion [60]. It also can produce obvious antitumor activity in human cancer patients. Antimitotic depsipeptides are receiving attention in cancer treatment as microtubule-targeted compounds. Tasidotin is a microtubule-targeted derivative of the marine depsipeptide dolastatin-15 and is undergoing clinical evaluation for cancer treatments. It inhibits proliferation of breast cancer cells with an adaptable dosage [61].

The hybrid compound cyclic hydroxamic acid-containing peptide (CHAP) is a unique way to develop isoform-specific HDAC inhibitors. CHAPs inhibit HDAC activity *in vivo* and affect gene expression. HDAC inhibition by CHAPs corresponds to natural cyclic tetrapeptide antibiotics. HDAC1 is highly sensitive to all CHAPs, much more than HDAC6 [62].

Apicidin[cyclo(*N*-*O*-methyl-l-tryptophanyl-l-isoleucinyl-d-pipecolinyl-l-2-amino-8-oxodecanoyl)] is a cyclic tetrapeptide with a potent broad spectrum of anti proliferative activity against various cancer cell lines. It effectively inhibits cell proliferation in ER-positive human breast cancer cells by altering the expression of cell cycle regulator proteins and inducing apoptotic cell death and induces the up-regulation of p53 [63]. The special cell-specific effects of apicidin are associated with ERα-mediated transcriptional regulation by the regulation of cell cycle arrest and apoptosis. After apicidin treatment in MCF-7 breast cancer cells, the expression of ERα and ERβ is decreased in a dose-dependent manner. Apicidin treatment obviously increases the levels in acetylated histone H3 and H4 in H-ras-transformed human breast epithelial (MCF10A-ras) and non-transformed epithelial (MCF10A) cells. MCF10A-ras cells showed a significantly higher growth rate than MCF10A cells on the anti-proliferative effects [64]. Apicidin also obviously decreases occupation of the first exon of the HSD17B1 gene by Polymerase II shown by chromatin immunoprecipitation analysis. It significantly downregulates the HSD17B1 transcript and protein in adenocarcinoma cells by repression of HSD17B1 gene transcription [65].

### 3.4. Benzamides

MS-27-275, one of the most active benzamide derivatives, causes hyperacetylation of nuclear histones in various tumor cell lines. It changes the cell cycle distribution, decreases the S-phase cells, and increases the G1-phase cells [66]. MS-275 (entinostat), a class I HDAC-selective inhibitor, has been used in clinical trials for cancer patients and is currently used in phase II trials. MS-275 can enhance radiosensitivity [67]. It can inhibit breast cancer tumor growth, angiogenesis, and metastasis. Through the involvement of both cell-extrinsic and cell-intrinsic pathways of apoptosis, MS-275 can sensitize TRAIL (tumor necrosis factor–related apoptosis-inducing ligand)-resistant breast cancer xenografts and can be combined with TRAIL to treat invasive breast cancer. MS-275 causes an accumulation of acetylated histones H3 and H4 in total cellular chromatin. MS-275 drives invasive breast cancer cells to undergo the reversal of EMT (epithelial-mesenchymal transition). By inducing epithelial cell markers, inhibiting mesenchymal cell markers, and regulating the expression of transcription factors, MS-275 ultimately leads to the suppression of cancer metastasis [68]. The combination of MS-275 with Adriamycin mediated by the transcription factor Sp1 to treat breast cancer cells can significantly increase apoptotic cell death by activation of both death receptor and mitochondrial apoptotic pathways [69].

CI-994(N-acetyl-dinaline), a novel oral histone deacetylase inhibitor, is a substituted benzamide derivative that has significant antitumor activity *in vitro* and *in vivo* against a broad spectrum of murine and human tumor models [70]. The mechanism of inhibition by CI-994 is not yet known. It causes accumulation of acetylated histones, although it is not able to inhibit HDAC activity in a direct fashion. CI-994 has been used in several Phase I studies and can be combined with other chemotherapeutic agents [71].

## 4. Conclusions

The use of epigenetic therapy, reversing the changes of DNA methylation and histone acetylation patterns, has large potential in the treatment of breast cancer. The recognition of epigenetics offers new avenues for drug discovery and therapeutics [72]. A synergistic effect of a combined use of DNMT and HDAC inhibitors has been observed. With the development of targeted inhibitors of epigenetic modifications, it provides the use of personalized targeted therapies. However, epigenetic drugs, leading to reactivation of tumor-suppressor genes, are critical to the normal functioning of cells. These drugs can be therapeutically used independently or in conjunction with other therapeutic modalities. The combination of conventional therapy with epigenetic therapy using DNMT inhibitors and HDAC inhibitors produces the optimal effect [73]. Currently, there has been little success in treating breast cancer by epigenetic drugs. But the drugs continue to undergo clinical trials despite the successful clinical uses of epigenetic therapies to treat hematological malignancies.
